# A Sputtered Silicon Oxide Electrolyte for High-Performance Thin-Film Transistors

**DOI:** 10.1038/s41598-017-00939-6

**Published:** 2017-04-11

**Authors:** Xiaochen Ma, Jiawei Zhang, Wensi Cai, Hanbin Wang, Joshua Wilson, Qingpu Wang, Qian Xin, Aimin Song

**Affiliations:** 1 School of Electrical and Electronic Engineering, University of Manchester, Manchester, M13 9PL United Kingdom; 2 Center of Nanoelectronics and School of Microelectronics, Shandong University, Jinan, 250100 China

## Abstract

Low operating voltages have been long desired for thin-film transistors (TFTs). However, it is still challenging to realise 1-V operation by using conventional dielectrics due to their low gate capacitances and low breakdown voltages. Recently, electric double layers (EDLs) have been regarded as a promising candidate for low-power electronics due to their high capacitance. In this work, we present the first sputtered SiO_2_ solid-state electrolyte. In order to demonstrate EDL behaviour, a sputtered 200 nm-thick SiO_2_ electrolyte was incorporated into InGaZnO TFTs as the gate dielectric. The devices exhibited an operating voltage of 1 V, a threshold voltage of 0.06 V, a subthreshold swing of 83 mV dec^−1^ and an on/off ratio higher than 10^5^. The specific capacitance was 0.45 µF cm^−2^ at 20 Hz, which is around 26 times higher than the value obtained from thermally oxidised SiO_2_ films with the same thickness. Analysis of the microstructure and mass density of the sputtered SiO_2_ films under different deposition conditions indicates that such high capacitance might be attributed to mobile protons donated by atmospheric water. The InGaZnO TFTs with the optimised SiO_2_ electrolyte also showed good air stability. This work provides a new pathway to the realisation of high-yield low-power electronics.

## Introduction

Oxide-semiconductor thin-film transistors (TFTs) have attracted much attention recently in industry and academia due to their high carrier mobility, low fabrication temperature, and low cost^1^. For applications such as displays, sensing devices, low-cost disposable electronics, portable electronics, and low-power electronics, it is highly desirable for TFTs to be capable of operating at low voltages^2, 3^. In order to achieve a low operating voltage of 1 V, a few-nm-thick gate dielectric layer can be used^4^. However, such a thin dielectric layer may cause a high leakage current as well as inhomogeneity issues in large-area electronics^5^. Alternatively, high-κ dielectrics can be employed to increase the gate specific capacitance and hence reduce the switching voltage of TFTs^6, 7^. However, they often have large fixed charge trap densities, resulting in poor threshold voltage control^8^ and current leakage problems^9, 10^.

Another interesting approach to achieving low-voltage operation is by using ionic liquids or ion gels as gate dielectrics, which can form electric double layer (EDL) with very high capacitance, typically more than 1 µF cm^−2^ at 1 Hz^11–13^. Such a high gate capacitance enables an extremely low TFT operating voltage, typically around 1 V. Despite this, the bottleneck issue is that the existing ionic liquids and gels are not suitable for industrial applications because of their soft and liquid nature and thereby the difficulty to control their shape and thickness in TFT structures. Polymer electrolytes or polyelectrolytes have also shown high specific capacitance^14, 15^. Such materials can be obtained in solid-state forms, but they usually exhibit poor chemical stability particularly at elevated temperatures^16^. Oxide-based solid-state electrolytes, such as porous SiO_2_ and Al_2_O_3_, have also emerged as an alternative to the conventional electrolytes recently^17–19^. Their porous structure offers a large effective surface area, capable of accommodating a large number of mobile protons^17–19^. Porous oxide films are normally produced by sol-gel synthesis which usually requires high-temperature annealing and results in poor uniformity^20, 21^. Plasma-enhanced chemical-vapour deposition (PECVD) has been used to fabricate oxide-based electrolytes at room temperature^17–19, 22, 23^. Sputtering is one of the most favourable deposition methods in electronics industry due to high film uniformity, low cost, and ease of large-area or even roll-to-roll film deposition at room temperature. However, to the best of our knowledge, there has been no report on sputtered solid-state electrolytes for TFTs to date.

In this work, we attempt to produce solid-state SiO_2_ electrolytes by sputtering technology for the first time. A range of Ar pressures and sputtering powers have been experimented during SiO_2_ sputtering, which result in very different SiO_2_ microstructures and therefore TFT behaviours. Under optimised sputtering conditions, InGaZnO (IGZO) TFTs that utilise the SiO_2_ electrolyte show an ultra-low operating voltage of 1 V, a near-zero threshold voltage, *V*_th_, of 0.06 V, a subthreshold swing, *SS*, of 83 mV dec^−1^ which is close to the theoretical minimum value, and a high on-off ratio of ~10^5^. The microstructure of the sputtered SiO_2_ electrolyte is characterised by scanning electron microscopy (SEM) and high-resolution transmission electron microscopy (HRTEM). The mass density of the sputtered SiO_2_ is determined by using Rutherford backscattering spectrometry (RBS). The EDL formation mechanisms have also been discussed based on studies of the ambient stability of the SiO_2_-electrolyte-based TFTs.

## Results

### DC and AC characteristics of EDL TFTs

**Figure 1:**
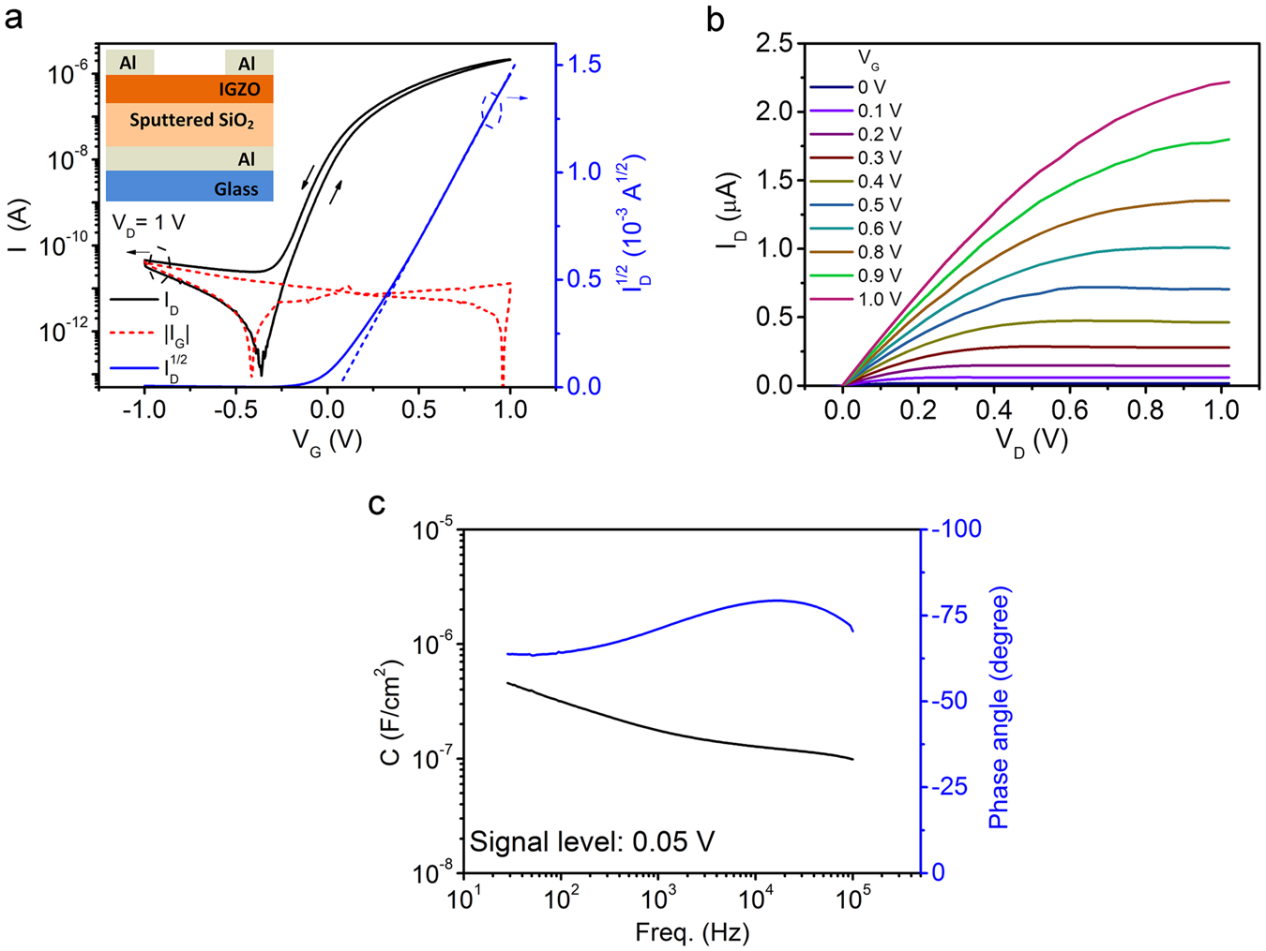
Characteristics of EDL TFTs. (**a**) Transfer characteristics of the TFT gated with SiO_2_ deposited at 85 W and an Ar pressure of 5 × 10^−3^ mbar on a glass substrate. The schematic cross-sectional view of the device is shown in the inset. The channel width and the channel length are 1.5 mm and 80 μm, respectively. (**b**) Output characteristics of the TFT. (**c**) Specific capacitance of the sputtered SiO_2_ layer as a function of frequency and the phase angles during the measurement.

Figure 1a shows the transfer characteristics of the TFT using a sputtered 200 nm-thick SiO_2_ layer as gate dielectric. The current on/off ratio and subthreshold swing are found to be ~10^5^ and 83 mV dec^−1^, respectively. In the saturation region, the threshold voltage is found to be very close to zero at 0.06 V. An anticlockwise hysteresis with a small threshold voltage shift of −0.02 V is observed at a sweep rate of 15 mV s^−1^. The total leakage current is found to be less than 0.1 nA and the leakage current density is around 6.0 × 10^−8^ A cm^−2^. Figure 1b shows the output characteristics of the TFT. A typical linear region is observed at low drain voltages. At a gate voltage, *V*_G_, of 1 V, and a drain voltage, *V*_D_, of 1 V, a drain current, *I*_D_, higher than 2 μA is obtained.

To demonstrate the stability and repeatability of device properties, the performance of ten TFTs fabricated in two batches at different times but under the same conditions were measured. Since the capacitance of electrolyte gate dielectric is a function of frequency (unlike conventional TFTs), we have chosen to analyse the transconductance *g*_*m*_ rather than mobility itself. A detailed statistical analysis of on/off ratio, subthreshold swing, threshold voltage and transconductance at *V*_G_ = 1 V and *V*_D_ = 2 V with the average value and standard deviation bar is shown in Supplementary Figure S1. Devices numbered 1 to 5 were fabricated in one batch and devices 6 to 10 were fabricated in another batch. The electrolyte dielectrics were deposited with an RF power of 85 W and an Ar pressure of 5 × 10^−3^ mbar. According to the statistical results, the devices fabricated in different batches showed similar performance. The on/off ratios of the devices are all around 3 × 10^5^ with a minimum value still higher than 1 × 10^5^. The magnitude of subthreshold swing is always better than 100 mV dec^−1^ with an average value of 85 mV dec^−1^. The transconductance values of the devices are also close to each other, indicating good uniformity and reproducibility.

It should be noted that our sputtered SiO_2_ electrolyte layer is a new type of dielectric to enable an extremely low operating voltage. Conventional SiO_2_ insulator has a relative dielectric constant of 3.9, meaning that a layer of 200-nm-thick SiO_2_ insulator provides a specific capacitance of around 17 nF cm^−2^. However, our 200 nm SiO_2_ electrolyte exhibited a specific capacitance of about 300 nF cm^−2^ which is more than one order of magnitude higher. Indeed, our IGZO TFTs showed an operating voltage of 1 V, which is a drastic improvement from more than 10 V operating voltage of TFTs gated with conventional SiO_2_ dielectric^24–26^. This is also significantly better than that of IGZO TFTs gated with 200-nm-thick high-κ dielectrics, such as Ta_2_O_5_ (3 V)^27^, HfO_2_ (5 V)^28^ and ZrO_2_ (6 V)^28^.

Figure 1c shows the specific capacitance of the 200 nm-thick SiO_2_ film at frequencies ranging from 20 Hz to 100 kHz using an Al/SiO_2_/Al sandwich structure. The low phase angle, smaller than −45°, indicates the sputtered SiO_2_ layer remains capacitive up to 100 kHz (ref. 29). A maximum capacitance of 0.45 µF cm^−2^ is obtained at 20 Hz, which is 26 times larger than that of thermally oxidised 200 nm-thick SiO_2_ (17.3 nF cm^−2^). Such a high capacitance enables the ultra-low operating voltage of the TFT in this work. Moreover, unlike thermally oxidised SiO_2_, the capacitance of the sputtered SiO_2_ shows a strong frequency dependence which is similar to the gate capacitance of other EDL transistors^17, 29, 30^.

### Effects of different sputtering conditions

**Figure 2:**
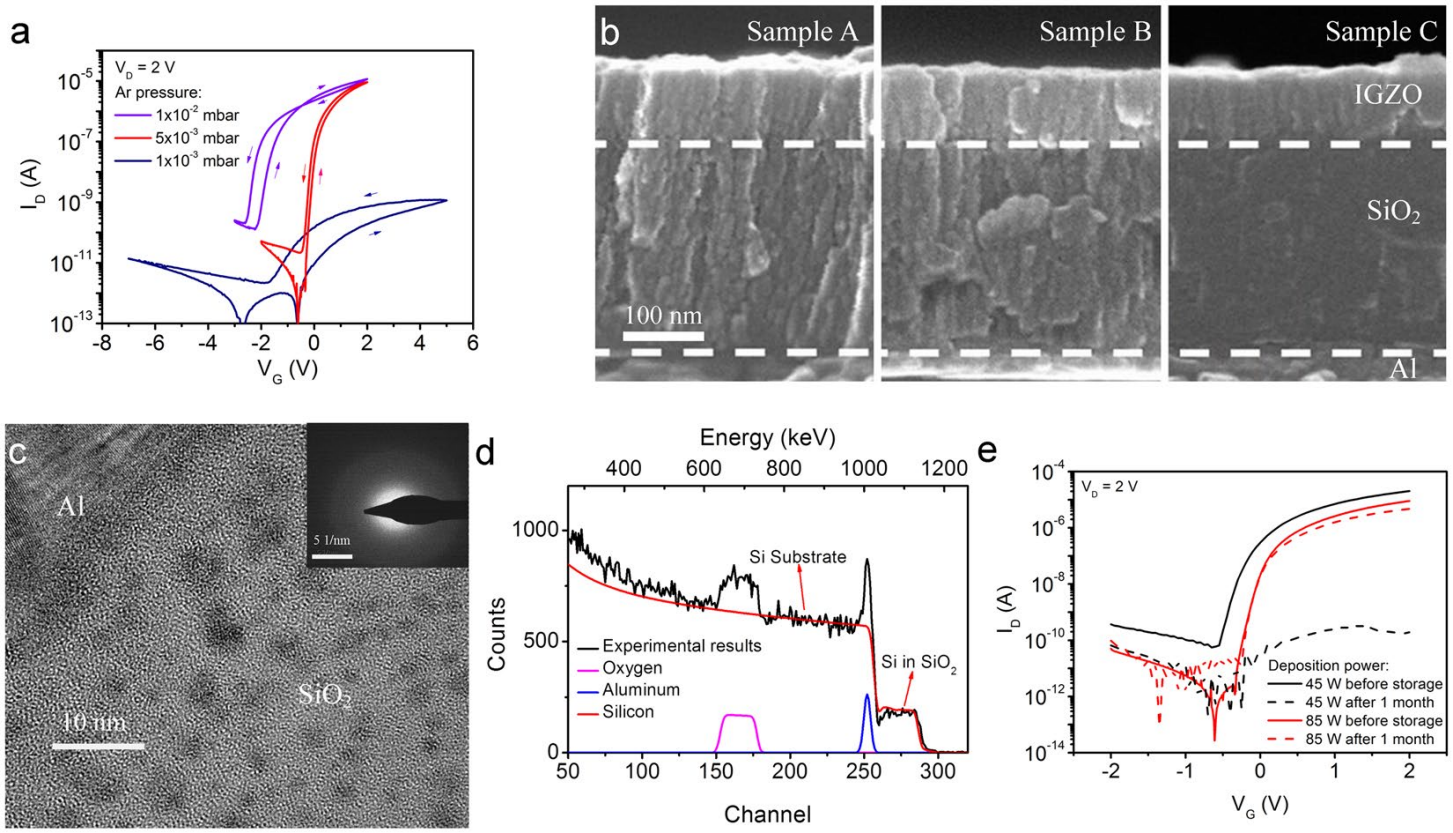
Effects of difference sputtering conditions. (**a**) Transfer characteristics of the IGZO TFTs based on the SiO_2_ gate dielectric sputtered at 85 W with Ar pressures of 1 × 10^−2^, 5 × 10^−3^, and 1 × 10^−3^ mbar, respectively. (**b**) High resolution cross-section SEM images of the TFTs with the SiO_2_ gate dielectric sputtered at Ar pressures of 1 × 10^−2^ (left, Sample A), 5 × 10^−3^ (middle, Sample B), and 1 × 10^−3^ mbar (right, Sample C), respectively. (**c**) Cross-sectional HRTEM bright field micrograph of the SiO_2_ layer. The SAED image of the SiO_2_ film is shown in the inset. (**d**) Rutherford backscattering spectrum of the SiO_2_ electrolyte deposited on Al/Si substrate at 85 W with an Ar pressure of 5 × 10^−3^ mbar. (**e**) Transfer characteristics of the TFTs using 45 W-sputtered and 85 W-sputtered SiO_2_ as gate dielectric after one month in atmospheric conditions.

In order to explore the origin of such high capacitance, a series of sputtering conditions have been experimented during the deposition of the SiO_2_ layer. Figure 2a shows the transfer characteristics of three IGZO TFTs gated with 200 nm-thick SiO_2_ dielectrics sputtered at different Ar pressures of 1 × 10^−2^, 5 × 10^−3^, and 1 × 10^−3^ mbar, respectively. The sputtering power is fixed at 85 W. The TFT with the SiO_2_ layer sputtered at 1 × 10^−3^ mbar shows a much lower on-current compared with the values obtained by the other two TFTs. An anticlockwise hysteresis is observed for all three devices, indicting mobile ions in the dielectric layer^31^. The TFT with the SiO_2_ layer sputtered at 1 × 10^−2^ mbar also shows a small region of clockwise hysteresis at gate voltages higher than −0.5 V, indicating electron trapping at the dielectric/channel interface^31^. Previous studies indicate that protons are common mobile ions in solid-state EDL transistors^29, 30, 32^. As the deposition process in this work does not involve any noticeable source of protons, it is plausible that they are introduced by moisture in the ambient air. It has been reported that water might be able to diffuse into the sputtered IGZO layer^33, 34^. Since the size of a water molecule is only around 2.5 Å (ref. 35), it is reasonable to assume that there might be some water molecules at the surface of, or inside, the SiO_2_ layer if the structure of SiO_2_ is porous.

According to Thornton’s model, increasing deposition pressure shall result in a reduction of deposition rate and even a porous film structure^36, 37^. Thus it is important to analyse the microstructures of the sputtered SiO_2_ films. Figure 2b shows the cross-sectional SEM images of these three TFTs, corresponding to the three different sputtering pressures, 1 × 10^−2^ (Sample A), 5 × 10^−3^ (Sample B), and 1 × 10^−3^ mbar (Sample C), respectively (Full images can be found in Supplementary Figures S2–4). It is found that there are more granular-like structures in Sample A than those in Sample B. There are hardly any granular-like structures in Sample C when the sputtering pressure is the lowest which is as expected. A highly granular-like microstructure is capable of absorbing water molecules and desirable for proton conduction^38, 39^. The absorbed water molecules may be ionised into H^+^ and OH^−^. When a positive bias is applied to the gate electrode, protons are repelled to the dielectric/channel interface^32^. These repelled protons will induce a large number of electrons in the channel, thus forming an EDL at the dielectric/channel interface. Because of the lack of granular-like microstructure in Sample C, the SiO_2_ layer sputtered at the lowest Ar pressure cannot absorb a significant amount of water to form an EDL, resulting in a much lower TFT current as shown in Fig. 2a.

Figure 2c shows an HRTEM image of the SiO_2_ layer sputtered at 5 × 10^−3^ mbar, which is obtained in bright field mode where a darker region indicates a higher material density. The SiO_2_ film exhibits an amorphous structure with an inhomogeneous density distribution. The selected area electron diffraction (SAED) image in the inset of Fig. 2c only shows a diffuse halo without clear rings or spots, confirming the amorphous structure of the material. These results suggest that the sputtered SiO_2_ has a low-density network structure surrounding higher density regions. Such low-density structure may promote proton hopping between oxygen atoms in the SiO_2_ layer^32, 40, 41^.

For thermally oxidised SiO_2_ films, the Si/O atom ratio and mass density are around 1:2.1 and 2.25 g cm^−3^, respectively^42, 43^. However, the RBS spectrum, as shown in Fig. 2d, indicates that the Si/O atom ratio and mass density for the SiO_2_ films sputtered at 85 W with an Ar pressure of 5 × 10^−3^ mbar are 1:2.7 and 1.87 g cm^−3^, respectively. The low mass density confirms that the sputtered SiO_2_ film at high Ar pressures has a porous structure. The small value of the Si/O ratio indicates the existence of excess oxygen atoms, suggesting that there are a large number of hydroxyl groups or water molecules at the surface of, or inside, the SiO_2_ layer.

The power dependence of the SiO_2_ electrolyte has also been studied. Figure 2e shows the transfer characteristics of two TFTs gated with dielectrics sputtered at the same Ar pressure of 5 × 10^−3^ mbar but different RF powers of 45 W (low power) and 85 W (high power), respectively. According to Messier’s model, the kinetic energy of sputtered particles from the target is positively correlated to the RF power^36, 37^. As such, at the lower RF power (45 W), the sputtered particles will have lower energy to self-organise to form a denser film on the substrate. The turn-on voltage of the lower-power-sputtered device is indeed lower than the value obtained for the higher-power-sputtered device as shown in Fig. 2e.

### Stability test

**Figure 3:**
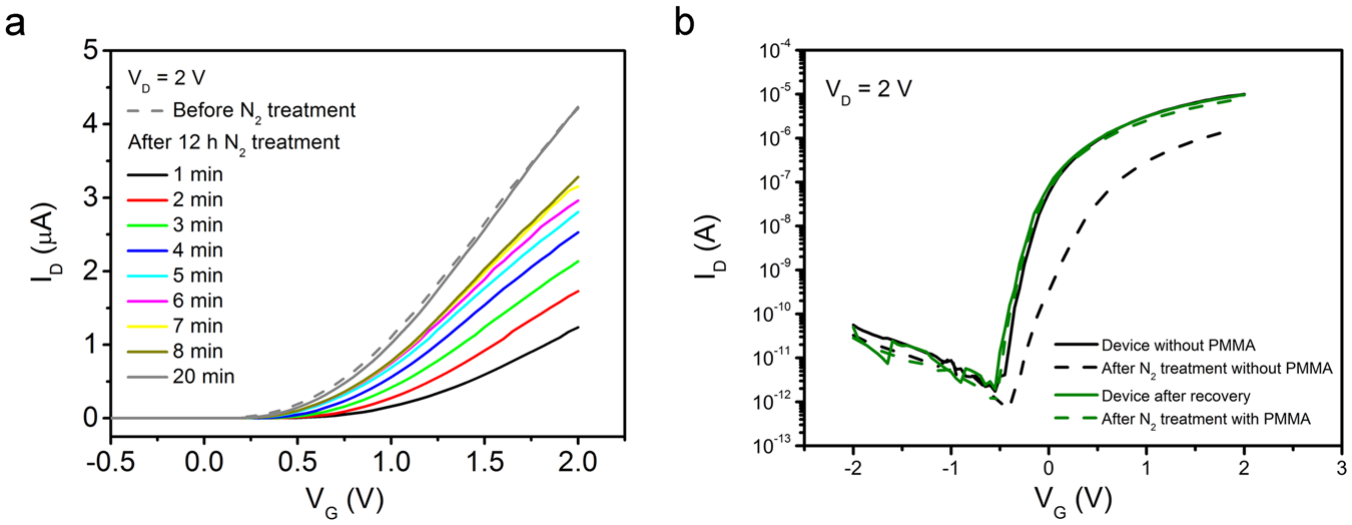
Stability of EDL TFTs. (**a**) Transfer characteristics of the TFT gated with 200-nm thick SiO_2_ sputtered at 85 W before (dashed line) and recovering from (solid lines) a treatment in dry N_2_ for 12 hours. (**b**) Transfer characteristics of IGZO EDL TFTs with and without a capping layer of 400 nm PMMA, before and after a dry N_2_ treatment for 12 hours.

As protons in the sputtered SiO_2_ film may be generated by ionised water molecules, testing the air stability of the SiO_2_ electrolyte TFTs may offer a deeper understanding of the EDL formation mechanism. As shown in Fig. 2e, the performance of the TFT with SiO_2_ electrolyte sputtered at the higher power (85 W) remains almost the same after one-month storage in ambient atmosphere. On the contrary, the transfer characteristic of the TFT with SiO_2_ electrolyte sputtered at the lower power (45 W) shows a significant degradation. However, the performance of the lower-power-sputtered device can be recovered after annealing for 1 h in N_2_ at 100 °C (shown in Supplementary Figure S5). The annealing treatment may remove the water molecules inside the electrolyte and restore the device performance. However, the exact mechanism that causes the very different ambient stabilities of the 45 W and 85 W devices is not clear and needs further studies. Figure 3a shows the transfer characteristics of the TFT with higher-power-sputtered SiO_2_ gate dielectric before and after treatment in dry N_2_ for 12 h. The drain current at a gate voltage of 2 V is 4.6 µA before the N_2_ treatment and slightly drops to 1.2 µA immediately after taking the device out of the N_2_ ambient. The drain current increases continuously after leaving the device in ambient atmosphere until the device regains its high performance after about 20 min. This confirms that the sputtered SiO_2_ electrolyte functions by absorbing water and suggests that protons are the mobile ions that are responsible for the formation of EDL layer.

It is common for electrolyte-based TFTs to be sensitive to the environment. Such a property itself can be useful in developing environmental sensors^44, 45^. Furthermore, it is possible to apply a capping layer to control the environmental stability of the devices. Here, we deposited a layer of 400 nm PMMA as a capping layer on some of the devices, and compared their environmental stability before and after PMMA capping. As shown in Fig. 3b, a device without PMMA capping shows a much lower on current after a dry N_2_ treatment for 12 hours due to the reduction of protons or hydrogen ions in the electrolyte. The TFT was then placed in ambient atmosphere for one hour, which resulted in a recovery in device performance as shown by the green curve. After capping the TFT by spin coating a 400 nm-thick PMMA layer, the device was placed in the dry N_2_ chamber for 12 hours and measured again. No clear degradation was observed as indicated by the dashed green curve. Such an experiment confirms that adding a capping layer can improve and control the stability of our TFTs, and the useful method is most likely applicable also to other types of EDL devices.

## Discussion

In summary, we report the first SiO_2_ solid-state electrolyte deposited by sputtering technology. TFTs based on the sputter ed SiO_2_ electrolytes exhibit high performance including 1 V operation, nearly ideal subthreshold swing, low leakage current and small hysteresis. Since sputtering technology is widely used in industry for large-area and even roll-to-roll film deposition, the solid-state electrolyte developed in this work could have timely implications in low-cost, low-power, portable electronics applications.

## Methods

### Device Fabrication

IGZO TFTs were fabricated with a bottom-gate top-contact structure using photolithography. Glass substrates were cleaned with deionised water, acetone, and methanol. Thermal evaporation was used to deposit a 30 nm-thick Al layer for use as the gate electrode. A 200 nm-thick SiO_2_ gate dielectric was deposited by using RF sputtering under various conditions in Ar from a SiO_2_ target. Then a 50 nm-thick IGZO film was deposited as the channel layer by RF sputtering at 45 W and an Ar pressure of 5 × 10^−3^ mbar. Finally, 30 nm-thick Al source/drain electrodes of the TFTs were thermally evaporated. To deposit a capping layer on the top of a device, a 400 nm-thick layer of PMMA was spin-coated onto the device from a 4% solution in anisole (950PMMA A4, diluted to 4% with anisole). A shadow mask based IGZO TFT, which requires no chemical process, was also fabricated to prove that the ions were not induced during the photolithography process. The transfer characteristics of such IGZO TFT are shown in Supplementary Figure S6.

### Device Characterisation

I-V characteristics of the IGZO TFTs were measured by using a Keysight E5270 semiconductor analyser at room temperature in dark. A Keysight E4980A LCR meter was used to measure the C-V characteristics. The high-resolution SEM images were obtained by using an FEI Nova NanoSEM 450 scanning electron microscope. The HRTEM analysis was performed by using a Tecnai F30 transmission electron microscope operating at 300 kV. The mass density and Si/O atom ratio were measured by using National Electrostatics Corporation (NEC) 5SDH-2 RBS with a 2 MeV He^2+^ ion beam in vacuum.

## Electronic supplementary material

Supplementary Information
