# Supplementary material for: A Sputtered Silicon Oxide Electrolyte for High-Performance Thin-Film Transistors
Source: Sci Rep. 2017 Apr 11;7:809. doi: 10.1038/s41598-017-00939-6 (PMC5429786; doi:10.1038/s41598-017-00939-6)
Supplement: Supplementary file 1 — Supplementary Information [file 41598_2017_939_MOESM1_ESM.doc]

A Sputtered Silicon Oxide Electrolyte for High-Performance Thin-Film Transistors

Xiaochen Ma^a^, Jiawei Zhang^a^, Wensi Cai^a^, Hanbin Wang^b^, Joshua Wilson^a^, Qingpu Wang^b^, Qian Xin^b^* and Aimin Song^ab^*

^a^School of Electrical and Electronic Engineering, University of Manchester,

Manchester M13 9PL, United Kingdom

^b^Center of Nanoelectronics and School of Microelectronics, Shandong University, Jinan 250100, China

^*^Correspondence and requests for materials should be addressed to Q.X. and A.S. (email: [xinq@sdu.edu.cn](mailto:xinq@sdu.edu.cn) and [A.Song@manchester.ac.uk](mailto:A.Song@manchester.ac.uk))

# Supplementary Information

**Supplementary Figure S1.** Statistical analysis of on/off ratio, subthreshold swing, threshold voltage and transconductance at *V*_G_=1 V and *V*_D_= 2 V shown with the average value and standard deviation bar for TFTs fabricated in two sets. Devices numbered with 1 to 5 were fabricated in one batch and devices 6 to 10 were fabricated in another batch. The electrolyte dielectrics was deposited with an RF power of 85 W and an Ar pressure of 5×10^-3^ mbar.


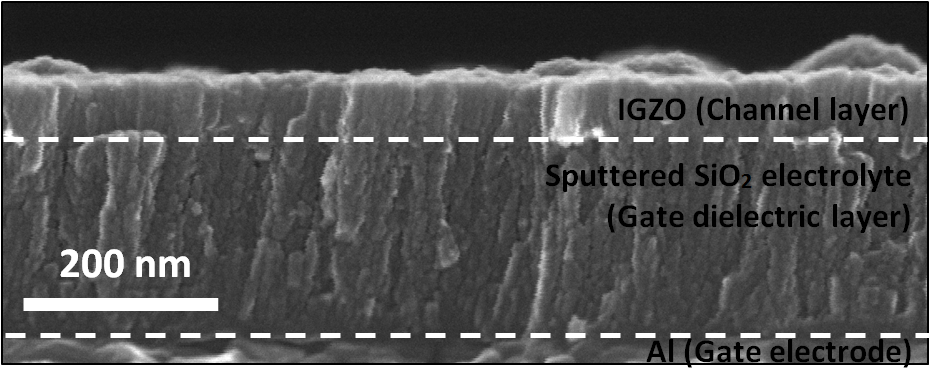


**Supplementary Figure S2.** Full-sized SEM image of sample A in Figure 2b. High-magnification cross-section SEM image of the EDL TFTs based on SiO_2_ electrolyte sputtered at the Ar pressure of 1×10^-2^ mbar with the deposition power of 85 W.


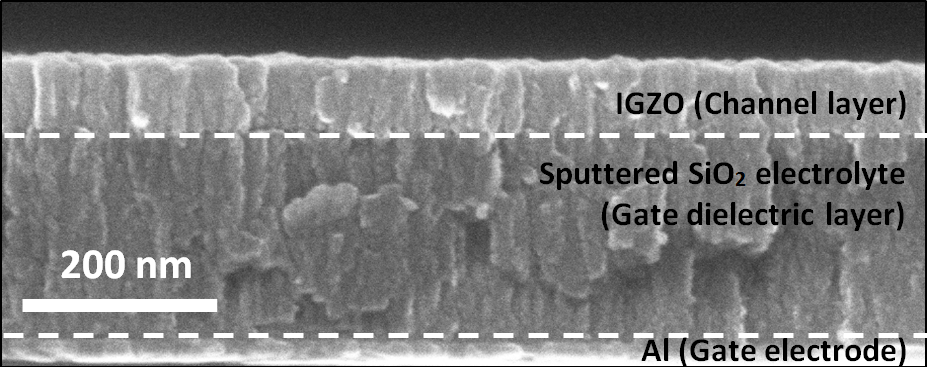


**Supplementary Figure S3.** Full-sized SEM image of sample B in Figure 2b. High-magnification cross-section SEM image of the EDL TFTs based on SiO_2_ electrolyte sputtered at the Ar pressure of 5×10^-3^ mbar with the deposition power of 85 W.


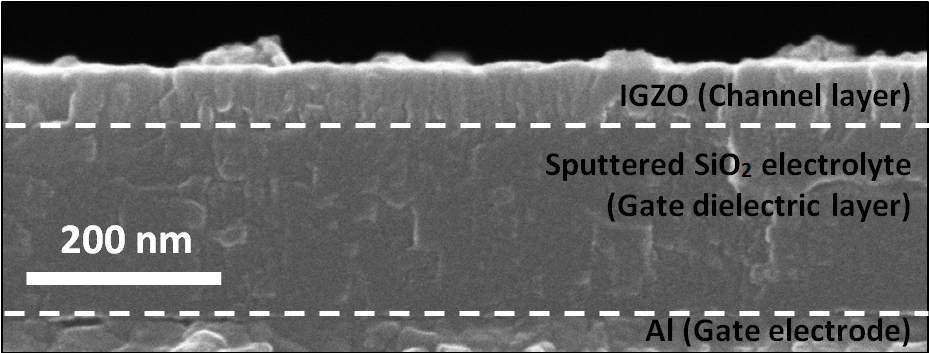


**Supplementary Figure S4.** Full-sized SEM image of sample C in Figure 2b. High-magnification cross-section SEM image of the EDL TFTs based on SiO_2_ electrolyte sputtered at the Ar pressure of 1×10^-3^ mbar with the deposition power of 85 W.

**
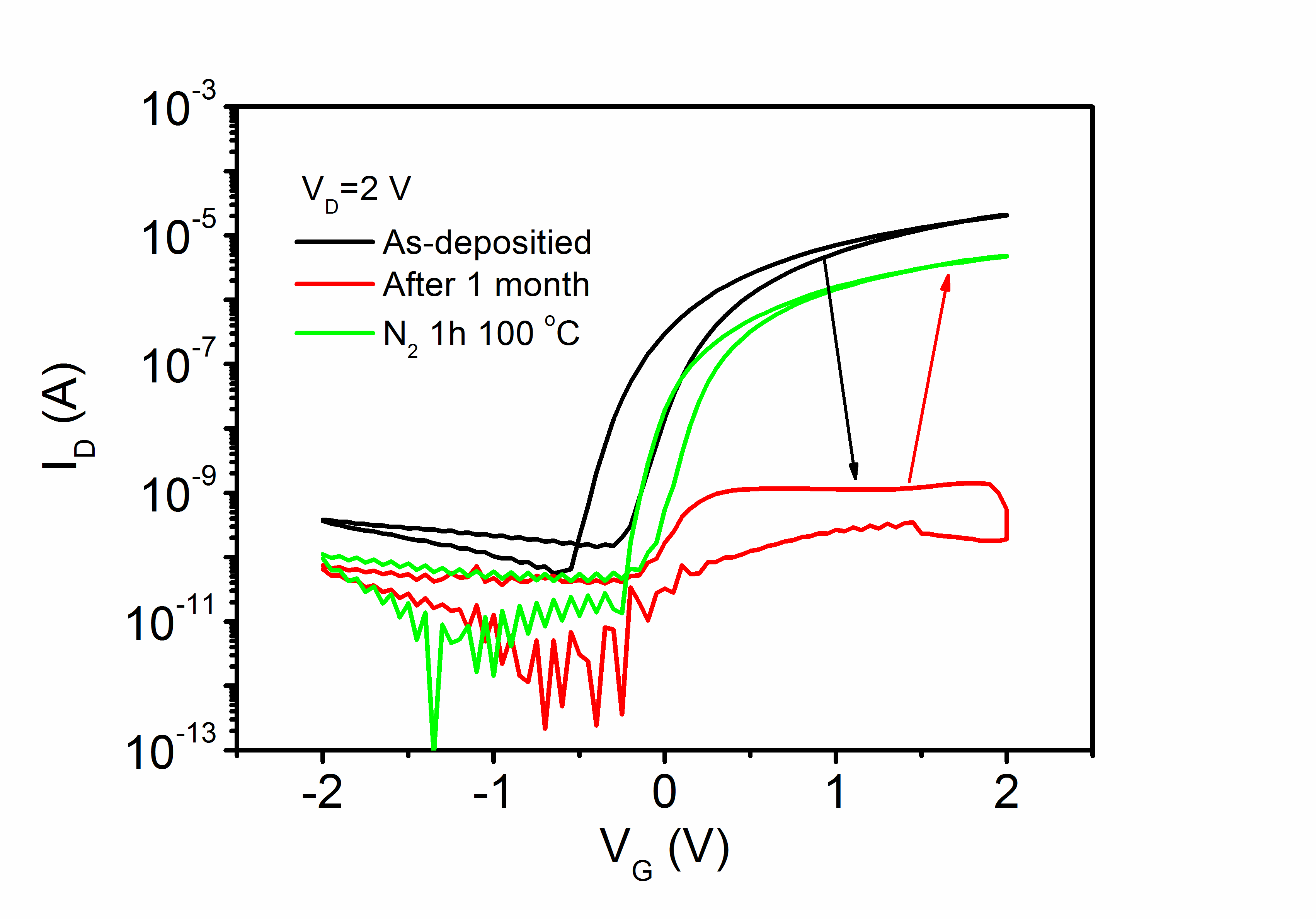
**

**Supplementary Figure S5.** Transfer characteristics of the TFTs with SiO_2_ sputtered at 45 W (black line), one month in air ambient (red line), and after annealing in N_2_ at 100 ^o^C for 1 h (green line).


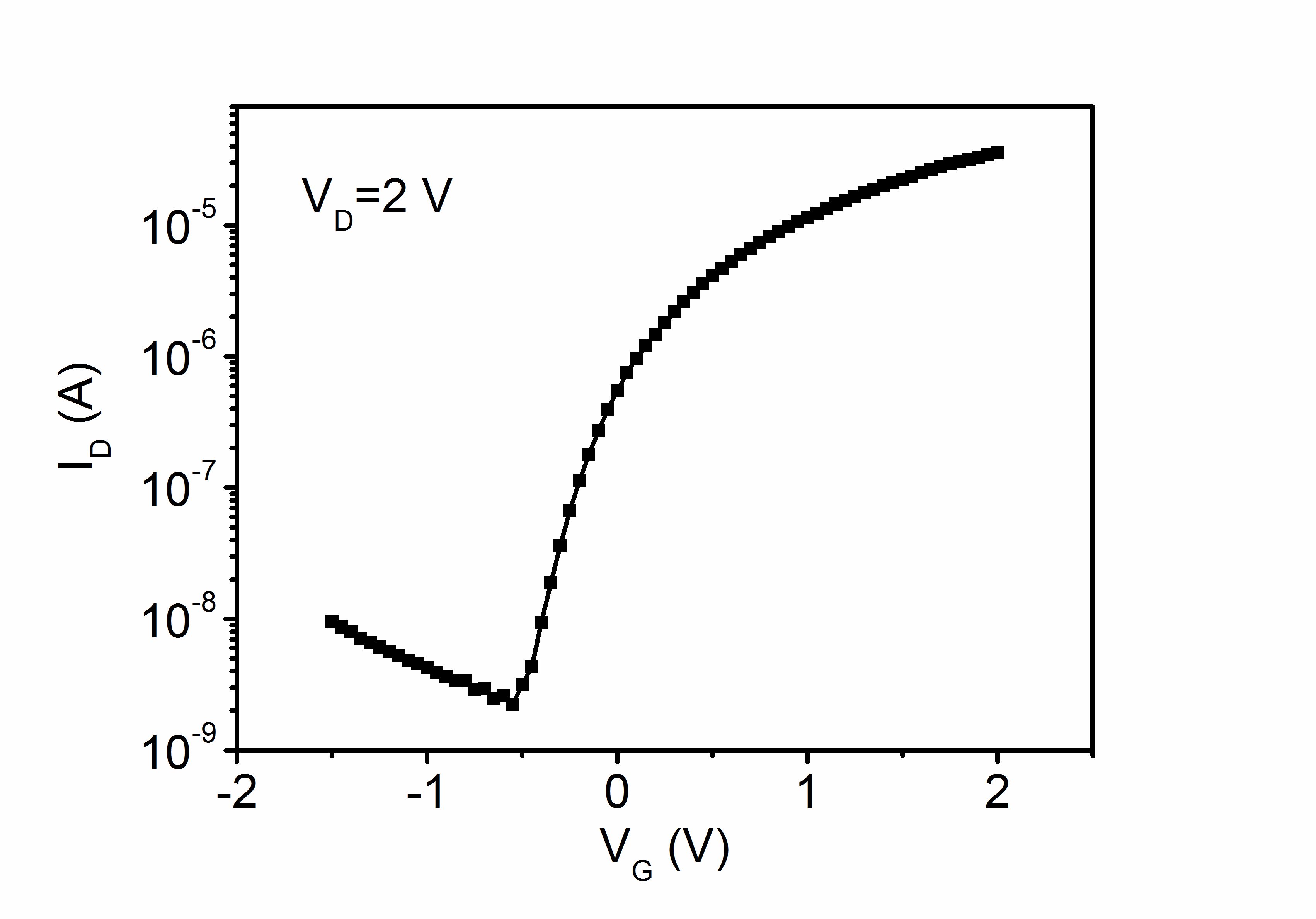


**Supplementary Figure S6.** Transfer characteristics of the TFTs using shadow mask for channel, source and drain. The channel length and width are 60 µm and 2 mm, respectively.
